# Size-confined fixed-composition and composition-dependent engineered band gap alloying induces different internal structures in _L_-cysteine-capped alloyed quaternary CdZnTeS quantum dots

**DOI:** 10.1038/srep27288

**Published:** 2016-06-02

**Authors:** Oluwasesan Adegoke, Enoch Y. Park

**Affiliations:** 1 Laboratory of Biotechnology, Research Institute of Green Science and Technology, Shizuoka University, 836 Ohya, Suruga-ku, Shizuoka 422-8529, Japan; 2 Laboratory of Biotechnology, Department of Bioscience, Graduate School of Science and Technology, Shizuoka University, 836 Ohya, Suruga-ku, Shizuoka 422-8529, Japan

## Abstract

The development of alloyed quantum dot (QD) nanocrystals with attractive optical properties for a wide array of chemical and biological applications is a growing research field. In this work, size-tunable engineered band gap composition-dependent alloying and fixed-composition alloying were employed to fabricate new _L_-cysteine-capped alloyed quaternary CdZnTeS QDs exhibiting different internal structures. Lattice parameters simulated based on powder X-ray diffraction (PXRD) revealed the internal structure of the composition-dependent alloyed Cd_x_Zn_y_TeS QDs to have a gradient nature, whereas the fixed-composition alloyed QDs exhibited a homogenous internal structure. Transmission electron microscopy (TEM) and dynamic light scattering (DLS) analysis confirmed the size-confined nature and monodispersity of the alloyed nanocrystals. The zeta potential values were within the accepted range of colloidal stability. Circular dichroism (CD) analysis showed that the surface-capped _L_-cysteine ligand induced electronic and conformational chiroptical changes in the alloyed nanocrystals. The photoluminescence (PL) quantum yield (QY) values of the gradient alloyed QDs were 27–61%, whereas for the homogenous alloyed QDs, the PL QY values were spectacularly high (72–93%). Our work demonstrates that engineered fixed alloying produces homogenous QD nanocrystals with higher PL QY than composition-dependent alloying.

Band gap engineering has been used as a valuable tool in the chemical tuning of semiconductor materials’ structures, especially at the nanoscale. Semiconductor quantum dot (QD) nanocrystals are characterized by their band gap properties, which can be tuned by altering their particle size^1^,^2^,^3^. The size-tunable optical properties of QDs constitute an attractive feature for different spheres of science and technology, including biolabeling, biosensing, optoelectronics, lasers and bioimaging^4^,^5^,^6^,^7^.

Another alternative method for tuning QDs semiconductor bandgap is by varying the metal chalcogenide composition via engineered control of the particle stoichiometry^7^,^8^. Different alloyed QD nanocrystals with ternary or quaternary structures have been produced via composition-dependent alloying^9^,^10^, and reports have shown that they possess superior output efficiency over conventional QD systems^11^,^12^,^13^. Tailoring the band gap of alloyed QDs without altering the particle size has been reported previously^14^. However, to date, this ability has only been used to mitigate the problem associated with extremely small (<2 nm) QD nanocrystals, which are unstable in device applications^15^. Therefore, leveraging the alloying properties of QDs coupled with their size-tunable feature is a unique way to obtain alloyed nanocrystals of different sizes and compositions.

For chemical and biological applications, the production of nanostructured systems with unique optical properties is urgently needed. In this work, we have synthesized, for the first time, water-soluble _L_-cysteine-capped alloyed quaternary CdZnTeS QDs via a hydrothermal aqueous synthetic route. The band gap alloying of CdZnTeS QDs was engineered using two fabrication techniques: (i) by employing a fixed composition of the metal chalcogenide to vary the particle size and (ii) by varying the composition of the Cd/Zn chalcogenide molar fraction to obtain QDs with different sizes and compositions. We employed a range of characterization techniques to study the structural and optical properties of the alloyed QDs produced via the two fabrication techniques. The results revealed that water-soluble _L_-cysteine-capped alloyed quaternary CdZnTeS QDs have great promise in biological and chemical applications.

## Results and Discussion

### Structural properties

#### Powder X-ray diffraction (PXRD)

**Figure 1:**
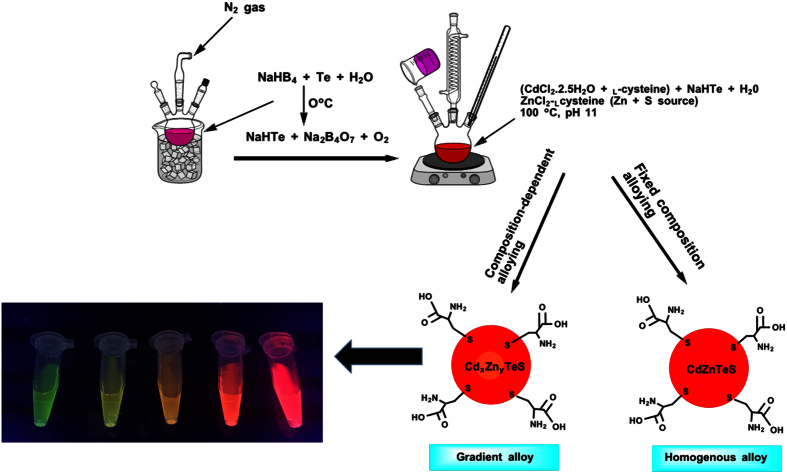
Schematic representation of the hydrothermal fabrication synthesis of the size-tunable composition-dependent and fixed-composition alloyed _L_-cysteine-capped CdZnTeS QDs.

**Figure 2:**
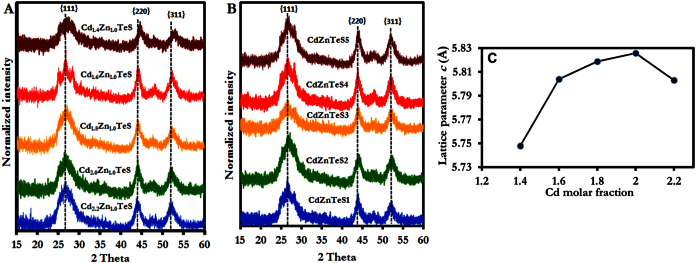
PXRD spectra of (**A**) composition-dependent and (**B**) fixed-composition alloyed _L_-cysteine-capped CdZnTeS QDs. Plot of the lattice parameters calculated from the PXRD data for alloyed _L_-cysteine CdZnTeS QDs with (**C**) different compositions.

Two fabrication techniques were employed to obtain _L_-cysteine-capped alloyed CdZnTeS QDs of different sizes (Fig. 1). PXRD was used to probe the internal structure of the alloyed QDs. Figure 2A shows the PXRD pattern of the size-tunable composition-dependent alloyed QDs. The XRD patterns clearly show that a zinc-blende crystal structure dominates both the composition-dependent and fixed-composition alloyed QDs. The XRD patterns are indexed to the {111}, {220} and {311} planes. The diffraction phase pattern and diffraction peak position are expected to be influenced by both the alloying process and the size-confined nature of the QDs. Based on the diffraction peak position in Fig. 2A, a slight shift to a higher Bragg angle occurred, as can be observed in the {220} and {311} planes. This slight shift in the peak position confirms that the QDs underwent the alloying process successfully. The XRD compositions were evaluated with respect to the Cd/Zn molar fractions and are listed in Table 1.

Figure 2B shows that the diffraction peak position of the size-tunable fixed-composition alloyed QDs did not display a significant shift to higher Bragg angle as the size increased. The diffraction peak position appears to be relatively in the same position. We speculate that the lack of peak shift in the QDs’ diffraction peak position is an indicator of the fixed-composition alloying process. It is important to emphasize that the diffraction pattern and peak position are not sufficient to characterize the natures of the internal structures of the alloyed QDs. Therefore, we plotted the lattice parameters of the alloyed QDs as a function of the Cd molar fraction for the composition-dependent QDs, which were determined the lattice parameter of the fixed composition alloyed QDs at different refluxing time. The lattice constants were simulated based on the diffraction patterns of the respective QDs. According to Vegard’s law, a linear relationship between the lattice parameter and the composition of solid-solution alloys indicates a homogenous alloy^16^. In contrast, nonlinearity reflects inhomogeneity, showing that the material has a gradient alloy structure. By analyzing the lattice parameter, a non-linear relationship was obtained for the size-tunable composition-dependent QDs (Fig. 2C). For the size-tunable fixed-composition QDs, the lattice parameter of the QDs obtained at different refluxing times (CdZnTeS1 → CdZnTeS5) was 5.83. The lack of change in the lattice parameter indicates a homogenous alloy. We tentatively conclude that the two fabrication techniques yielded alloyed QD nanocrystals with different internal structures: Composition-dependent alloying produced Cd_x_Zn_y_TeS QDs with a gradient structure, and the fixed-composition alloying produced CdZnTeS QDs with a homogenous structure.

#### Transmission electron microscopy (TEM)

**Figure 3:**
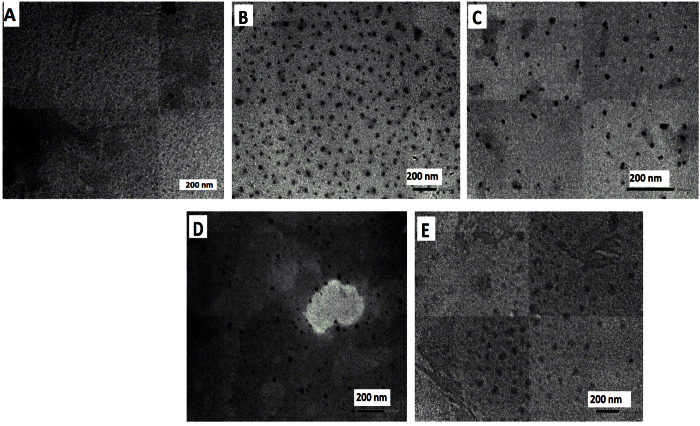
TEM images of the gradient alloyed _L_-cysteine (**A**) Cd_2.2_Zn_1.0_TeS, (**B**) Cd_2.0_Zn_1.0_TeS, (**C**) Cd_1.8_Zn_1.0_TeS, (**D**) Cd_1.6_Zn_1.0_TeS and (**E**) Cd_1.4._Zn_1.0_TeS QDs.

Figure 3A–E shows the TEM images of the gradient alloyed Cd_x_Zn_y_TeS QDs fabricated with different compositions based on the stoichiometric molar fraction of Cd/Zn. The shapes of the alloyed nanocrystals are nearly spherical in each TEM monograph, and the particles are monodisperse. In Fig. S1 (supplementary information), the TEM images of the homogenous alloyed CdZnTeS QDs are presented. We observe that the shape and dispersity pattern of the particles are nearly identical to those of the gradient alloyed QDs. In the TEM images of the gradient and homogenous alloyed QDs, it is difficult to observe any notable differences in the particles’ shapes and dispersities. However, we can infer that both the gradient and homogenous alloyed QDs display high degrees of monodispersity. It is important to emphasize that in this context, the term “homogenous” does not refer to the nature of the nanocrystals’ internal structures, as discussed in the XRD section, but instead relates to how the growth pattern of the QDs influences their dispersity. Interestingly, we observed a size-tunable feature with respect to the growth of the gradient and homogenous alloyed QDs. From Table 1, the particle size of the gradient composition-dependent QDs ranged from 3.1–8.2 nm, whereas that of the homogenous fixed-composition QDs ranged from 4.4–7.8 nm (Table 2). The tunable emission colors of the alloyed QDs shown in Fig. 1 indicate that these nanocrystals will be useful for chemical and biological applications.

#### Dynamic light scattering (DLS)

**Figure 4:**
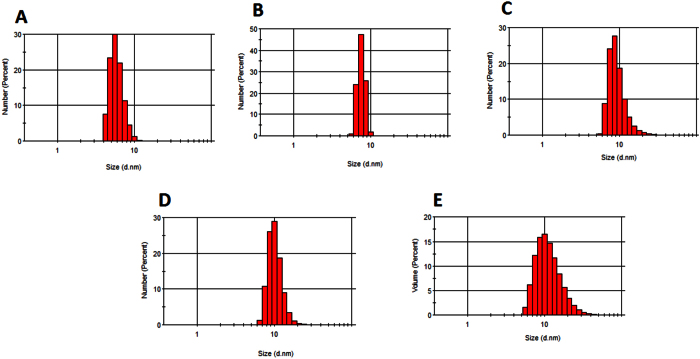
DLS hydrodynamic size curves of the gradient alloyed _L_-cysteine (**A**) Cd_2.2_Zn_1.0_TeS, (**B**) Cd_2.0_Zn_1.0_TeS, (**C**) Cd_1.8_Zn_1.0_TeS, (**D**) Cd_1.6_Zn_1.0_TeS and (**E**) Cd_1.4._Zn_1.0_TeS QDs.

DLS is an important tool for characterizing the size of nanoparticles in solution and determining their level of dispersity. Measurements were collected using scattered light from a laser, which penetrated through a colloidal solution of the alloyed QDs. The modulation of the scattered light intensity was analyzed to observe the hydrodynamic size of the QDs. The measured hydrodynamic size was also used to probe for particle agglomeration. If the alloyed QDs were agglomerated and exhibited a high polydispersity index, the measured DLS hydrodynamic size would be far larger than that determined via TEM. In contrast, if the QDs were unagglomerated, the DLS size would be similar to or slightly larger than the TEM size. For polydispersed QDs, a suspension of agglomerated nanoparticles has a DLS size in the range of 100–300 nm. Figures 4A–E and S2 (supplementary information) display the size-tunable DLS curves of the gradient and homogenous alloyed QDs, respectively. In Table 1, the DLS hydrodynamic size of the gradient alloyed QDs ranged from 6.0 to 11.9 nm while the standard deviation of the sizes ranged from 0.9–4.9 nm, whereas the DLS size of the homogenous alloyed QDs ranged from 0.7 to 5.7 nm (Table 2) while the standard deviation of the sizes ranged from 0.09–1.9 nm. It is evident from the DLS curves that a wider hydrodynamic size distribution is being exhibited by some of the QD nanocrystals. For example, the DLS curves for gradient Cd_1.4_Zn_1.0_TeS QDs (Fig. 4E), and homogenous CdZnTeS2 and CdZnTeS3 QDs (Fig. S2B,C) display a broader hydrodynamic size distribution. The reason for this may be due to the varying degree of dispersity of the particles in solution. Also, since the hydrodynamic size of the alloyed nanocrystals are very small (<20 nm), very little light is being scattered by the highly absorbing nanocrystals which thus influences the overall size distribution of the QDs in solution. Generally, the values based on the hydrodynamic size data are similar to those obtained from the TEM images. This verifies that both the gradient and homogenous alloyed QD nanocrystals are monodisperse and further confirms our TEM-based observations. Interestingly, the DLS sizes of the homogenous alloyed QDs are slightly lower than their TEM values, unlike the gradient alloyed QDs. We believe that this size variation is because of the nature of the QDs’ internal structures.

#### Zeta potential

**Figure 5:**
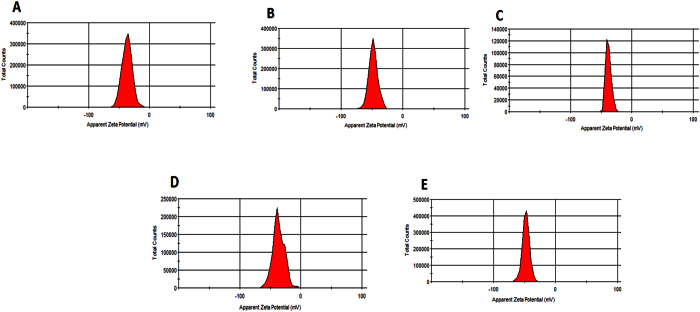
Zeta potential curves of the gradient alloyed _L_-cysteine (**A**) Cd_2.2_Zn_1.0_TeS, (**B**) Cd_2.0_Zn_1.0_TeS, (**C**) Cd_1.8_Zn_1.0_TeS, (**D**) Cd_1.6_Zn_1.0_TeS and (**E**) Cd_1.4._Zn_1.0_TeS QDs.

Nanoparticles generally have charged surfaces that attract thin layers of oppositely charged ions. As a nanoparticle diffuses through a solution, the double layers of ions move with it. The electric potential at the double-layer boundary is called the zeta potential and is typically in the range of −100 mV to +100 mV. The magnitude of the zeta potential is commonly used as a control and predictive measure to ascertain the long-term stability of the colloidal nanoparticle. Nanoparticles with zeta potential values lower than −30 mV or greater than +30 mV are considered to have greater degrees of stability^17^. Figure 5A–E, shows the zeta potential curves of the size-dependent gradient alloyed QDs, and Fig. S3 (supplementary information) shows the zeta potential curves for the size-dependent homogenous alloyed QDs. The presence of peak splitting in some of the curves is due to the steric repulsion of the surface capped _L_-csyteine ligand in solution. We believe the variation in the peak split may be influenced by the varying number of _L_-cysteine ligand anchored to the QDs surface. The zeta potential values for the gradient alloyed QDs are in the range of −36.9 mV to −48.3 mV (Table 1), whereas those for the homogenous alloyed QDs are in the range of −17.6 mV to −48.5 mV (Table 2). The gradient alloyed QDs’ high colloidal stability is demonstrated by their zeta potential values, which are lower than −30 mV. Except for homogenous CdZnTeS1 QDs, the alloyed QDs had zeta potential values within the range of colloidal stability.

### Optical properties

#### Circular dichroism (CD)

**Figure 6:**
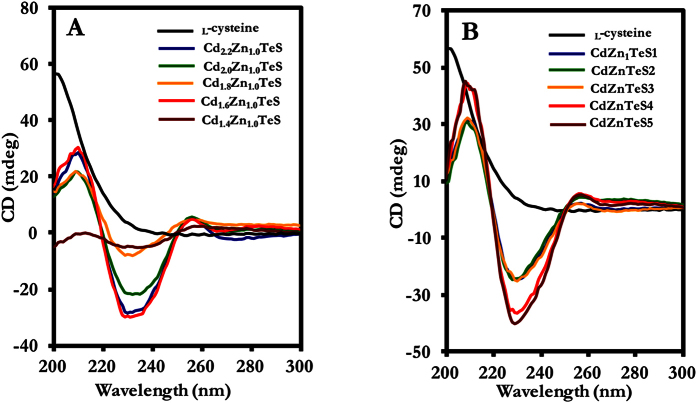
CD spectra of the (**A**) gradient composition-dependent and (**B**) homogenous fixed-composition alloyed _L_-cysteine CdZnTeS QDs.

Chirality is one of the most fascinating phenomena in the natural world^18^. Chiral compounds play a vital role in the fields of medicine, biology, pharmacology and chemistry^19^. Furthermore, the selection of chiral compounds is essential for disease therapy and diagnosis. In our study, we probed the chiral properties of the size-tunable _L_-cysteine-capped gradient and homogenous alloyed CdZnTeS QDs by CD. The CD spectra of the size-dependent gradient and homogenous alloyed QDs are shown in Fig. 6A,B, respectively. The CD spectra reveal two positive CD signals at approximately 210 nm and 257 nm and a negative CD signal at approximately 233 nm for both the gradient and homogenous alloyed QDs. Notably, no CD signal was observed for free _L_-cysteine ligand within the region of signals generated by the alloyed QDs. We believe that the emergence of CD signals in the alloyed QDs is attributable to changes in their electronic state and conformational structure induced by the surface-capped _L_-cysteine ligand. The chiral peaks at 210 nm and 233 nm for homogenous alloyed CdZnTeS QDs are much narrower than those of the gradient alloyed QDs, and we assume that this finding provides direct evidence regarding the nature of their internal structure. Elucidating the CD activities of the alloyed QD nanocrystals is complex, but we can generally infer that the CD signals are independent of the nanocrystal size. In contrast, the peak widths are dependent on the nature of the QD structure. We conclude that the alloyed QDs exhibit chiral properties, making them useful for biomedical applications.

#### Cd_x_Zn_y_TeS with gradient internal structure

**Figure 7:**
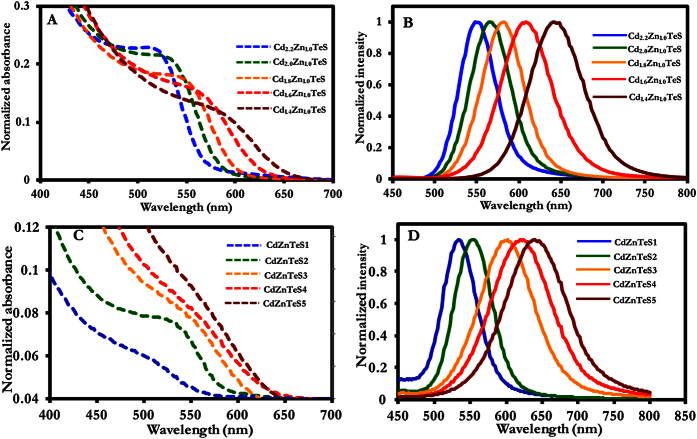
Evolution of the UV/vis and fluorescence emission spectra of alloyed CdZnTeS QDs of different sizes obtained by varying the Cd/Zn molar fraction (**A**,**B**) and using a fixed molar fraction (**C**,**D**).

The colloidal growth of the size-tunable gradient alloyed Cd_x_Zn_y_TeS QDs was monitored with respect to the temporal evolution of their optical properties. The temporal evolution of the absorption and photoluminescence (PL) emission spectra is indicative of the growth kinetics and formation of the alloyed nanocrystals. Various synthetic parameters, such as the precursor concentration, precursor material and metal molar fraction, influence the growth of QDs. In this section, we address how the Cd/Zn feed molar ratio affects the optical properties of the alloyed QDs. Alloyed size-tunable Cd_x_Zn_y_TeS QDs were prepared by varying the stoichiometric feed molar ratio of Cd_x_/Zn_y_, where x = 2.2, 2.0, 1.8, 1.6 and 1.4 and y = 1.0. Generally, each feed molar ratio corresponds to QDs with a particular particle size and PL emission wavelength. Thus, QDs’ band gap energies (Table 1) can be engineered by varying the Cd/Zn molar fraction. From the temporal evolution of the absorption and PL emission spectra shown in Fig. 7A,B, respectively, we observed a size-tunable spectral red shift with PL emission wavelengths specific to each alloy composition. Evaluating each of the PL emission spectra, we observe a dominant band edge PL emission that ultimately suppressed deep trap emission. The photophysical properties with respect to the absorption wavelength, PL emission wavelength and PL quantum yield (QY) are summarized in Table 1. As the QDs grew, the absorption wavelength red-shifted from 516 to 580 nm, and the PL emission wavelength was tuned from 548 to 642 nm. It is important to emphasize that the absorption and PL emission wavelengths of the QDs depend on their composition. Thus, the size of the QDs is specific to each alloy composition.

The PL QY is an important optical parameter that is generally used to determine the quality of QD nanocrystals and obtain information about the nature of their surfaces. PL QY as high as 61% was obtained for alloyed Cd_1.8_Zn_1.0_TeS QDs, approximately two-fold higher than that (27%) obtained for Cd_1.6_Zn_1.0_TeS QDs. As shown in Table 1, the PL QY ranged between 27 and 61%. Interestingly, the QY values are independent of the QDs’ size but are specific for each alloy composition. Thus, based on the PL QY variation, the composition-dependent alloying process induced surface passivation or surface defect states, which were sandwiched between radiative or nonradiative exciton recombination states. Because the alloying process was conducted from Cd_2.2_Zn_1.0_TeS → Cd_1.4_Zn_1.0_TeS, the declining QYs for Cd_2.0_Zn_1.0_TeS and Cd_1.6_Zn_1.0_TeS constitute evidence of nonradiative exciton recombination. However, effective surface passivation is evident in Cd_1.8_Zn_1.0_TeS and Cd_1.4_Zn_1.0_TeS. The PL QY variation can be attributed to a number of reasons. First, we can assume that the alloyed QDs are subjected to surface tension. According to the analysis of the lattice parameter determination (Fig. 2C), the value steadily increased until Cd_1.6_Zn_1.0_TeS and declined slightly for Cd_1.4._Zn_1.0_TeS QDs. This decline can be attributed to surface-induced tension^20^. Second, electron distribution and the cation-anion bond length may affect surface reconstruction^21^,^22^. Third, the charge distribution and cation-anion bond length could affect the surface ligand effect^21^. We believe these combined effects significantly affected the QY resulting from the alloying process.

Additionally, the dramatic decline in the QY for Cd_1.6_Zn_1.0_TeS is quite surprising. Generally, the photophysical properties of the composition-dependent alloy QDs are specific for each alloy composition. Therefore, a change in the intrinsic photophysical properties of the alloy QDs with respect to the significant decline in the PL QY can be influenced by strain relaxation and development^23^,^24^. This can be attributed to differences in lattice mismatch among the different metal chalcogenide materials. The strain relaxation effects results in optical band gap variations, such as the lack of a linear dependence of the PL QY as a function of the alloy composition. With the lack of an optical bowing effect, the alloyed QDs were found to be effective for band gap variation between 2.14 and 2.65 eV in a non-linear lattice behavior, hence producing a gradient internal structure.

#### CdZnTeS with homogenous internal structures

Unlike the fabrication of gradient alloyed Cd_x_Zn_y_TeS QDs, in which the Cd/Zn feed molar ratio was used to tune the optical properties, the optical properties of homogenous CdZnTeS QDs were tuned by employing a fixed molar composition of the metal chalcogenide material. As shown in Table 2, the band gap of homogenous alloyed CdZnTeS QDs could be engineered according to the growth of the QDs, indicating a quantum-confined nature. A fixed molar composition of Cd_2.2_Zn_1.0_TeS was selected to tune the absorption and PL emission spectra of the QDs. We used this molar composition because it facilitated effective QD nucleation and growth with high PL efficiency. Five different sizes were obtained using the same QD composition over time. Here, we aimed to study how the fabrication step influences the overall optical properties of the alloyed QDs. Figure 7C,D shows the size-tunable absorption and PL emission spectra of the QDs. The PL emission spectra of the QDs reveal that a band edge type of PL emission spectra dominates, with no appearance of deep trap emission. As shown in Table 2, the absorption wavelength was tuned from 510 to 570 nm, whereas the PL emission spectra were tuned from 534 to 638 nm. Careful inspection of the absorption spectra shows differences in the width of the excitonic peak. Except for CdZnTeS1 and CdZnTeS2, the excitonic peaks of the QDs broadened as they increased in size. The remarkable feature of this class of QDs is that their PL QY values are 72–93%. From a scientific perspective, these values are remarkably high and result from the QDs’ effective passivating surfaces. The highest PL QY obtained for the gradient alloyed QD was 63%, which was marginally lower than the lowest QY value (72%) obtained for the homogenous alloyed QDs. Significant suppression of the nonradiative exciton recombination state was generally achieved by employing a fixed alloy composition to tune the band gap of the QDs. Interestingly, we found that CdZnTeS2 and CdZnTeS3 QDs of different sizes exhibited the same PL QY value (93%). QDs of different sizes (in a single batch) typically do not possess the same PL QY value. In this case, however, dangling bonds, which create trap sites on the QD surface, were ultimately suppressed in the alloyed QDs engineered via quantum confinement.

Our results demonstrate that by employing a fixed composition of the metal chalcogenide material, the optical properties of alloyed CdZnTeS QDs can be tuned to achieve high PL efficiency. Multiple PL emission wavelengths were generated for a single alloy composition, and this technique will be useful when specific sizes are required for material applications, such as in multiplex detection and in biological imaging. It is also reasonable to assume that the chemical reactivity of the cadmium monomer toward zinc, tellurium and sulfur is fairly similar. Indeed, if the chemical reactivity were dissimilar, it would be difficult to create a homogenous alloy.

#### Optical bowing effect

**Figure 8:**
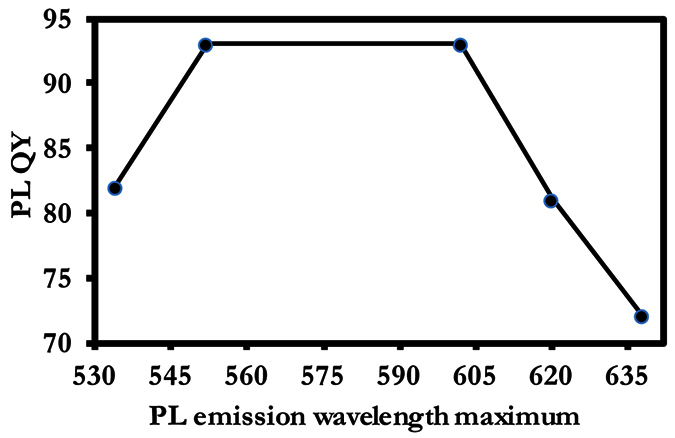
Plot of the PL QY of homogenous alloyed CdZnTeS QDs as a function of the PL emission wavelength maximum.

Further quantitative probing of the optical properties of the fixed-composition alloyed CdZnTeS QDs revealed the effect of optical bowing. Figure 8 shows the nonlinear optical bowing effect with respect to the relationship between the PL QY values of homogenous alloyed CdZnTeS QDs as a function of the PL emission wavelength maximum. Previous optical bowing effect has been observed in ternary alloyed CdSeTe QDs^25^. We believe the nonlinear optical bowing effect is induced by the size-confined fixed composition nature of the alloyed QD nanocrystals. Theoretically, Zunger and coworkers^26^,^27^ proposed that the nonlinear effect is caused by three electronic and structural phenomenon: (i) different electronegativity values are exhibited by the metal ions, (ii) different atomic sizes are exhibited by the different metal ions in the alloy structure, and (iii) different lattice constants are exhibited by the binary structures. It is surprising to observe an optical bowing effect in the homogenous CdZnTeS QDs, considering that the bond length mismatch and displacement of the metal cation are expected to be lesser than in the gradient CdZnTeS QDs, when taking into consideration the PL QY values.

## Conclusions

Fixed-composition and composition-dependent alloying techniques were employed to engineer the band gaps of novel water-soluble alloyed _L_-cysteine-capped CdZnTeS QD nanocrystals. The internal structures of the alloyed nanocrystals were determined by simulating their lattice parameters based on PXRD data. The results showed that composition-dependent alloying produced a gradient internal structure, whereas fixed-composition alloying yielded a homogenous internal structure. The monodispersity of the nanocrystals was confirmed by TEM and DLS analysis, and their colloidal stability was confirmed by zeta potential analysis. The nanocrystals’ chiroptical properties were confirmed using CD. In general, the photophysical data showed that the PL QY was higher for the homogenous alloyed QDs than for the gradient alloyed QDs.

## Methods

### Materials

Tellurium powder (Te), sodium borohydride (NaBH_4_), cadmium chloride hemipentahydrate, (CdCl_2_·2.5H_2_O), zinc chloride (ZnCl_2_), _L_-cysteine, rhodamine 6G, were purchased from Sigma Aldrich Co. LLC. (St. Louis, MO, USA). Ethanol and chloroform were purchased from Wako Pure Chemical Ind. Ltd. (Osaka, Japan). An ultra-pure Milli-Q Water System was used as the water source.

#### Synthesis of alloyed quaternary CdZnTeS QDs

_L_-cysteine-capped alloyed quaternary CdZnTeS QDs were synthesized via the conventional method for the hydrothermal aqueous-phase synthesis of QDs^28^ with modifications. First, the NaHTe precursor was prepared in ice under a constant flow of inert gas by mixing 0.85 g of Te powder and 0.53 g of NaHB_4_ with 20 mL of ultrapure Millipore water. The Cd precursor was prepared under an inert atmosphere by dissolving 1.0 g of CdCl_2_·2.5H_2_O and 1.2 g of _L_-cysteine in ultrapure Millipore water (100 ml) and adjusting the pH of the solution to 11 with 1.0 M NaOH. Freshly prepared NaHTe solution was then added into the Ar-saturated Cd precursor solution, followed swiftly by the addition of 20 mL of the ZnS precursor solution, which had been previously prepared by dissolving 0.27 g of ZnCl_2 _and 0.48 g of _L_-cysteine in 100 ml of Millipore water. _L_-cysteine was used as both the capping agent and sulfur source for the preparation of the ZnS precursor. The reaction was then heated to ~110 °C, and alloyed CdZnTeS QDs of different sizes but a fixed concentration were obtained at different time intervals. The alloyed QDs with different compositions and sizes were prepared in different batches by varying the stoichiometric molar fraction of Cd/Zn in the reaction mixture. A four-step procedure was employed to purify the alloyed QDs, as follows. Step 1: ethanol; Step 2: ethanol + chloroform; Step 3: ethanol; and Step 4: chloroform. Using the two fabrication steps, QDs with five different sizes were obtained. The different-sized composition-dependent alloyed QDs are denoted as Cd_2.2_Zn_1.0_TeS, Cd_2.0_Zn_1.0_TeS, Cd_1.8_Zn_1.0_TeS, Cd_1.6_Zn_1.0_TeS and Cd_1.4_Zn_1.0_TeS. The size-tunable alloyed QDs with a fixed composition are denoted as CdZnTeS1, CdZnTeS2, CdZnTeS3, CdZnTeS4 and CdZnTeS5. Figure 1 shows the schematic representation of the synthetic process used to fabricate the alloyed QDs.

#### Characterization

UV/vis absorption and fluorescence emission measurements were performed using a filter-based multimode microplate reader (Infinite^®^ F500, TECAN, Ltd., Männedorf, Switzerland). TEM images were obtained using a TEM JEM-2100F, (JEOL, Ltd., Tokyo, Japan) operated at 100 kV. PXRD measurements were collected using a RINT ULTIMA XRD (Rigaku Co., Tokyo, Japan) with a Ni filter and a Cu-Kα source. Data were collected from 2 theta = 5–60° at a scan rate of 0.01°/step and 10 s/point. Fourier transform infrared spectroscopy (FT-IR) analyses were carried out using a FT-IR (ATR 8700, Shimadzu Co., Tokyo, Japan). Zeta potential and DLS analyses were conducted using a zetasizer Nano series (Malvern Inst. Ltd., Malvern, UK). CD analyses were performed with a JASCO spectrophotometer (J-820 model, JASCO, Tokyo, Japan). The CD spectra were obtained at ambient temperature using a 1-mm quartz cell.

## Additional Information

**How to cite this article**: Adegoke, O. and Park, E. Y. Size-confined fixed-composition and composition-dependent engineered band gap alloying induces different internal structures in _L_-cysteine-capped alloyed quaternary CdZnTeS quantum dots. *Sci. Rep.* **6**, 27288; doi: 10.1038/srep27288 (2016).

## Supplementary Material

Supplementary Information

## Figures and Tables

**Table 1:** Summary of the structural and optical properties of the size-tunable composition-dependent alloyed _L_-cysteine Cd_x_Zn_y_TeS QDs.

**QDs (Cd/Zn molar fraction)**	**XRD (Cd/Zn composition)**	**PL λ**_**max**_ **(nm)**	**Abs λ**_**max**_ **(nm)**	**PL QY (%)**	**TEM** **size (nm)**	**DLS** **(nm)**^a^	**Zeta (mV)**^b^	**Band gap (eV)**
Cd_2.2_Zn_1.0_TeS	Cd_0.3_Zn_1.0_TeS	548	516	50	3.1	6.0 (1.2)	−37.6 (8.1)	2.65
Cd_2.0_Zn_1.0_TeS	Cd_0.5_Zn_0.3_TeS	566	530	40	5.0	7.6 (0.9)	−48.3 (7.0)	2.47
Cd_1.8_Zn_1.0_TeS	Cd_0.1_Zn_0.7_TeS	582	548	61	5.4	9.5 (3.0)	−38.8 (4.5)	2.36
Cd_1.6_Zn_1.0_TeS	Cd_0.3_Zn_0.8_TeS	608	562	27	6.3	10.4 (2.6)	−36.9 (9.5)	2.24
Cd_1.4_Zn_1.0_TeS	Cd_0.2_Zn_0.8_TeS	642	580	47	8.2	11.9 (4.9)	−48.2 (5.7)	2.14

^a^Standard deviation (nm).

^b^Standard deviation (mV).

**Table 2:** Summary of the structural and optical properties of the size-tunable alloyed _L_-cysteine CdZnTeS QDs with a fixed molar composition.

**QDs**	**PL λ**_**max**_ **(nm)**	**Abs λ**_**max**_ **(nm)**	**PL QY (%)**	**TEM size (nm)**	**DLS (nm)**^**a**^	**Zeta (mV)**^**b**^	**Band gap (eV)**
CdZnTeS1	534	510	82	4.4	0.7 (0.09)	−17.6 (8.6)	2.47
CdZnTeS2	552	526	93	5.1	1.4 (0.3)	−48.5 (9.0)	2.36
CdZnTeS3	602	540	93	5.5	5.2 (1.9)	−46.8 (9.2)	2.28
CdZnTeS4	620	554	81	5.8	5.3 (1.2)	−36.3 (9.2)	2.26
CdZnTeS5	638	~570	72	7.8	5.7 (1.4)	−42.0 (8.1)	2.20

^a^Standard deviation (nm).

^b^Standard deviation (mV).
