# Supplementary material for: Size-confined fixed-composition and composition-dependent engineered band gap alloying induces different internal structures in L-cysteine-capped alloyed quaternary CdZnTeS quantum dots
Source: Sci Rep. 2016 Jun 2;6:27288. doi: 10.1038/srep27288 (PMC4890122; doi:10.1038/srep27288)
Supplement: Supplementary Information [file srep27288-s1.pdf]

Size-confined fixed-composition and composition-dependent engineered band gap alloying induces different internal structures in L-cysteine-capped alloyed quaternary CdZnTeS quantum dots

Oluwasesan Adegoke, Enoch Y. Park[a,b]

[a] *Laboratory of Biotechnology, Research Institute of Green Science and Technology, Shizuoka University, 836 Ohya, Suruga-ku, Shizuoka 422-8529, Japan*

[b] *Laboratory of Biotechnology, Department of Bioscience, Graduate School of Science and Technology, Shizuoka University, 836 Ohya, Suruga-ku, Shizuoka 422-8529, Japan*

E-mail:
adegoke.sesan@mailbox.co.za (OA)
park.enoch@shizuoka.ac.jp (EYP)

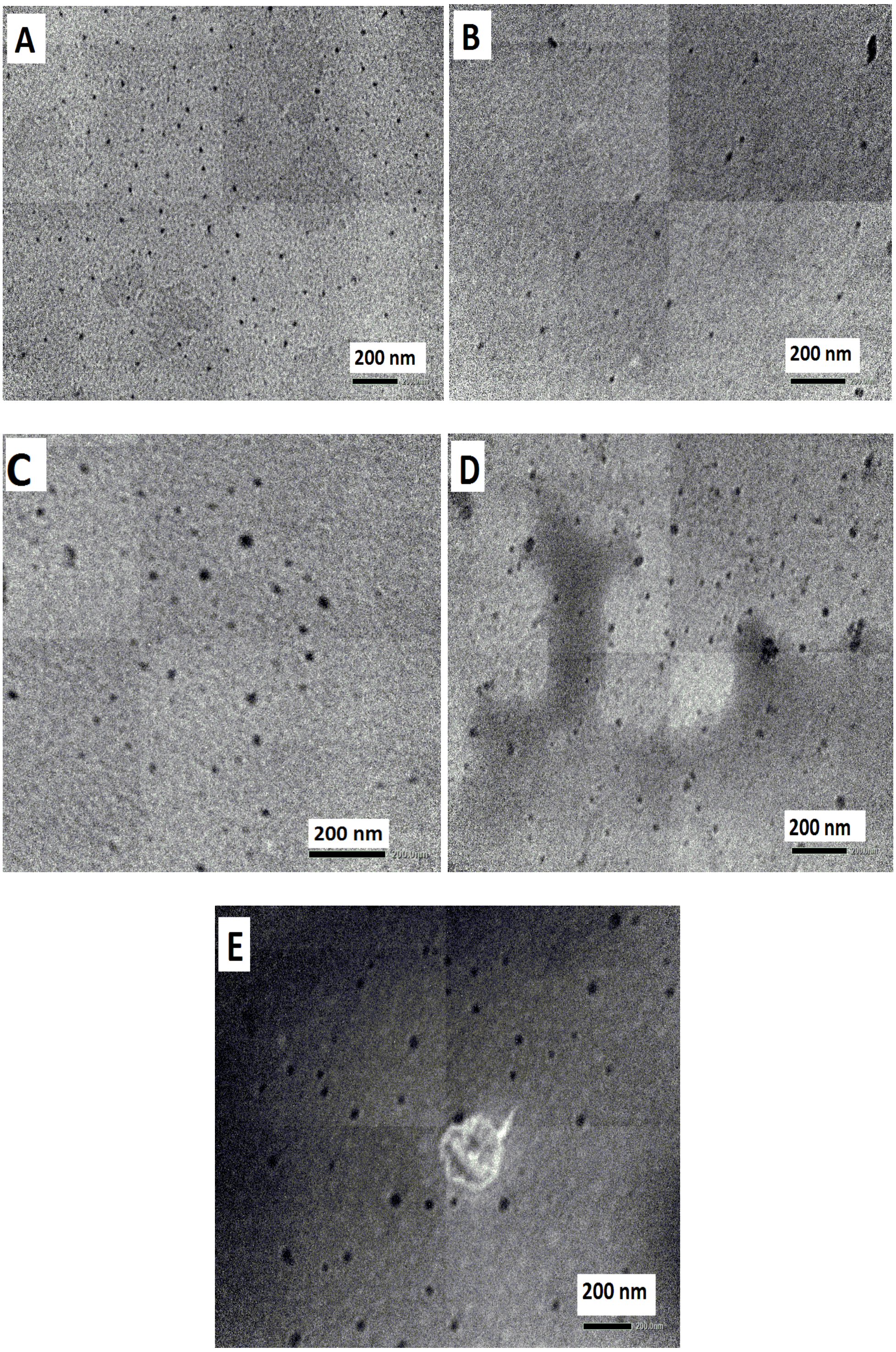

Fig. S-1. TEM images of alloyed L-cysteine (A) CdZnTeS1, (B) CdZnTeS2, (C) CdZnTeS3, (D) CdZnTeS4 and CdZnTeS5 QDs.

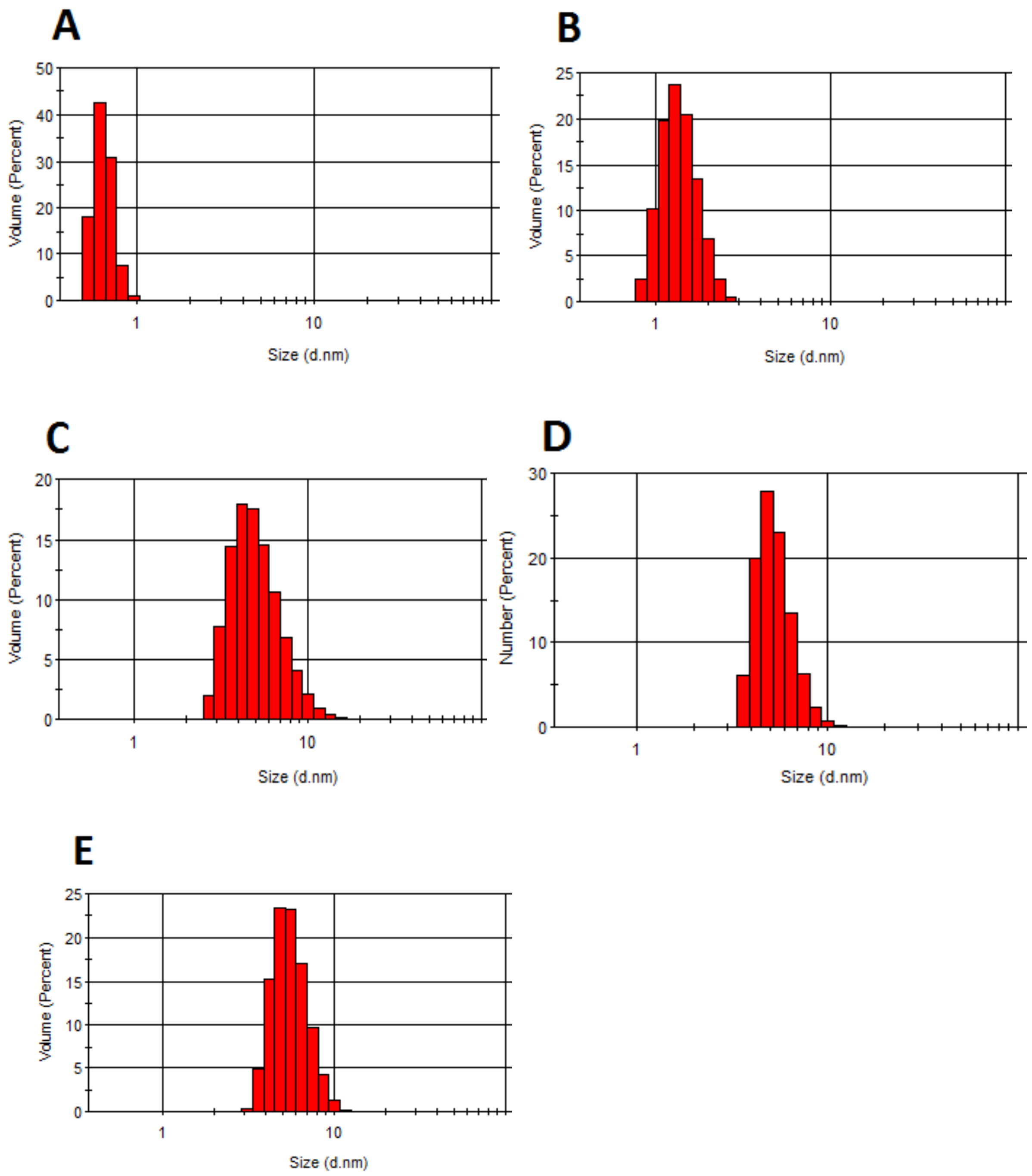

Fig. S-2. DLS hydrodynamic size curves of homogenous alloyed L-cysteine (A) CdZnTeS1, (B) CdZnTeS2, (C) CdZnTeS3, (D) CdZnTeS4 and CdZnTeS5 QDs.

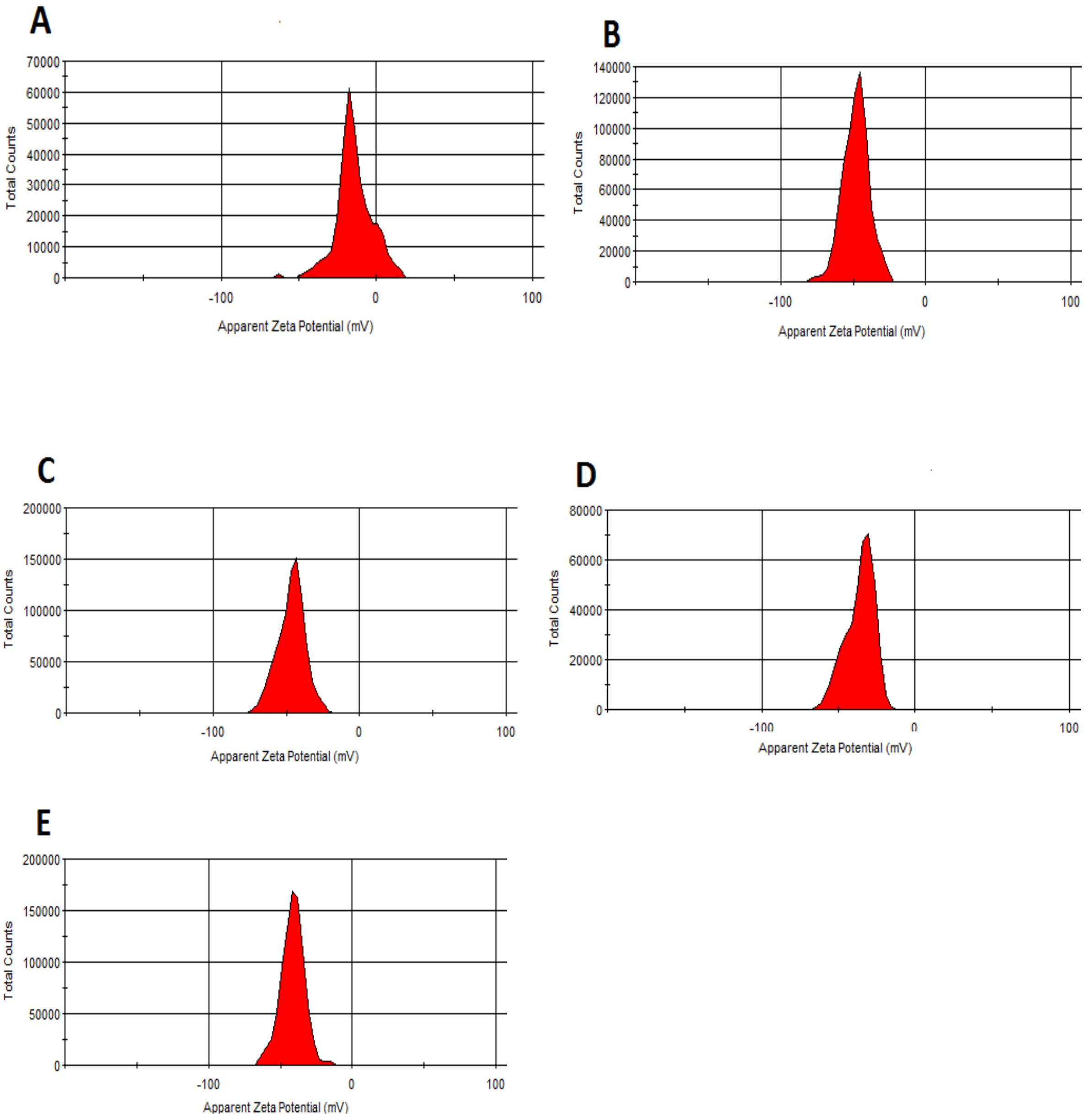

Fig. S-3. Zeta potential curves of homogenous alloyed L-cysteine (A) CdZnTeS1, (B) CdZnTeS2, (C) CdZnTeS3, (D) CdZnTeS4 and CdZnTeS5 QDs.
